# Case Report: Management of a patient with Sheehan’s syndrome presenting with anxiety and depression

**DOI:** 10.3389/fendo.2025.1487375

**Published:** 2025-04-28

**Authors:** Hongxia Liu, Rensheng Chen, Chengzhen Feng, Di Zhu, Chao Luo, Guanming Chen, Yuehua Chen

**Affiliations:** ^1^ Department of General Practice, Affiliated Jinhua Hospital, Zhejiang University School of Medicine, Jinhua Municipal Central Hospital, Jinhua, China; ^2^ Department of Orthopedic, Affiliated Jinhua Hospital, Zhejiang University School of Medicine, Jinhua Municipal Central Hospital, Jinhua, China

**Keywords:** Sheehan’s syndrome, hormone, anxiety, depression, case report

## Abstract

Sheehan’s syndrome is a rare and under-recognized disorder that may be encountered in a variety of clinical settings. The diagnosis of the condition is challenging due to its rarity and the fact that it is often overlooked in differential diagnoses. In addition, there are no characteristic clinical features or routine laboratory tests that immediately inform the diagnosis. We report the case of a 50-year-old woman diagnosed with an anxiety-depressive state, who had marked and progressive symptoms, which resolved more rapidly following treatment with escitalopram oxalate and recurred after discontinuation of the medication. Further examination revealed abnormalities in hormone levels and pituitary augmentation MRI, based which the patient was finally diagnosed with Sheehan’s syndrome. This case emphasizes the rarity of patients with Sheehan’s syndrome presenting with anxiety and depression, thereby presenting a diagnostic challenge to clinicians. The objective of this case report is to emphasize the necessity for heightened awareness among healthcare professionals regarding patients with rare clinical presentations and the significance of timely and collaborative treatment strategies.

## Background

Hypopituitarism is a clinical syndrome caused by damage to the hypothalamic-pituitary axis by various causes, which leads to insufficient secretion of one or more adenopituitary hormones. The degree of hypopituitarism varies from complete loss of all pituitary hormones (panhypopituitarism) to selective deficiency of specific hormones. Sheehan’s syndrome is a rare form of hypopituitarism. This condition arises from the ischemic necrosis of the anterior pituitary gland in peripartum women. In 2011, a study conducted in Iceland found that the incidence of Sheehan’s syndrome was 5.1 per 100,000 women (1). These patients tend to have a history of postpartum hemorrhage, but the severity of postpartum hemorrhage is not related to the severity of clinical manifestations.

The time for diagnosis of Sheehan’s syndrome is unclear. Some studies have indicated that the average time to diagnosis is 13 years after delivery (2, 3), with case reports demonstrating that some patients have been diagnosed as late as 30 years after childbirth (4). The symptoms of Sheehan’s syndrome can be acute or latent. Patients with undiagnosed Sheehan’s syndrome may initially be relatively asymptomatic, but symptoms may become apparent when confronted with a stressful state that exerts pressure on their thyroid and adrenal glands (5). Frequently, diagnosis is delayed because of atypical symptoms. The initial clinical manifestations of Sheehan’s syndrome are amenorrhea and agalactia, while other clinical manifestations include malaise, fatigue, chills, lethargy, hypotension, hypoglycemia, dry skin, and anemia. In addition, Sheehan’s syndrome can also be manifested as anxiety and depression (6–8). Neuropsychiatric manifestations in patients with hypopituitarism are uncommon, and there are few reports of hypopituitarism patients presenting with anxiety and depression.

The relationship between Sheehan’s syndrome and anxiety and depression is unclear. It is possible that abnormalities in the autoimmune and endocrine systems may be implicated. Anxiety and depression resulting from Sheehan’s syndrome are amenable to treatment, underscoring the importance of screening suitable patients to prevent long-term morbidity. This study presents a case of anxiety and depression associated with Sheehan’s syndrome, offering a valuable case study for further investigation.

## Case

The patient is a 50-year-old woman who had received only junior high school education and had pursued a career in agriculture for many years. She had been experiencing a range of mental health problems, which included frequent bouts of crying and a tendency to overthink, with her concerns primarily focused on her health and the challenges she was facing within her family. Of particular note, she displayed significant anxiety about her daughter’s inability to find a suitable boyfriend and the persistent financial difficulties faced by her family, which not only exacerbated her psychological burden, but also diminished her hope for the future. She exhibited excessive tension, persistent worry, and a profound sense of worthlessness, with these symptoms severely affecting her daily life and work efficiency. Furthermore, she exhibited a significant reduction in her sense of purpose in life and the emergence of suicidal ideation. Following a consultation at the mental health department, she was diagnosed with an anxiety-depressive state according to the DSM-5 diagnostic criteria and prescribed citalopram. After one month of treatment, the patient reported a partial recovery and ceased taking the drug citalopram. However, half a month later, the same symptoms reappeared, and the patient was referred to our inpatient unit for further evaluation of their condition.

Following an inquiry into the patient’s medical history, it was established that the patient was 27 years of age at the time of her first delivery This delivery was complicated by severe postpartum hemorrhage, which required the administration of substantial blood products and resulted in the patient’s admission to the intensive care unit to ensure stabilization of her vital signs. Subsequently, she encountered agalactia, amenorrhea, and infertility. Seven years later, she began experiencing intolerance to cold, fatigue, excessive sleepiness, frequent nightmares, dizziness, slowed thinking, and dry skin, all of which had progressed over the past 16 years. She denied substance abuse and any family history of mental illness. A physical examination revealed stable vital signs, edema in the limbs and face, dry skin, clear consciousness, correct orientation, low mood, presence of negative thoughts, no negative behaviors, tension, worry, and fear of dying. No abnormalities were found in the neurological examination.

**Figure 1:**
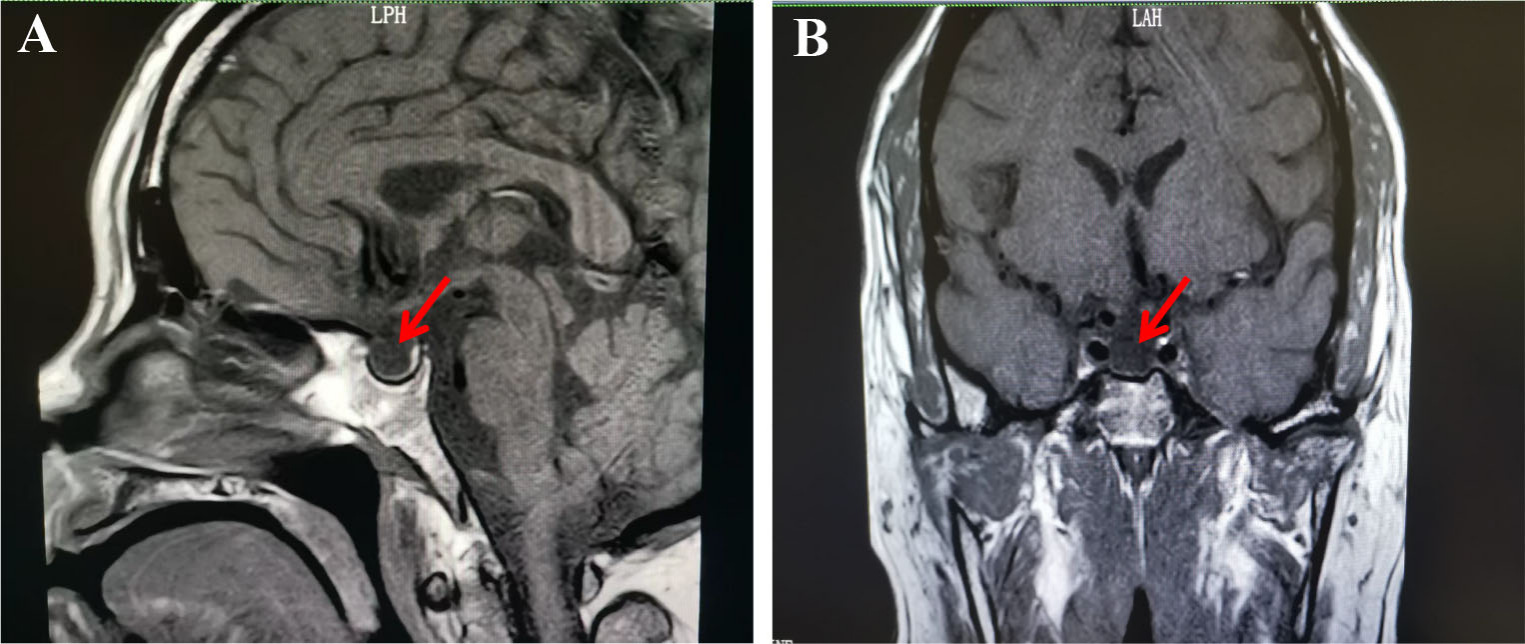
Enhanced pituitary magnetic resonance imaging shows empty sella.T1-weighted sagital **(A)** and coronal **(B)** magnetic resonance imaging reveal the sella filled with cerebrospinal fluid (red arrow).

In consideration of the patient’s clinical manifestations and signs, which exhibited similarity to those associated with Sheehan’s syndrome, hormonal levels and pituitary enhanced MRI were tested. The laboratory test results are outlined below: ① Thyroid hormones: FT4 <3.20 pmol/L, FT3 3.83 pmol/L, T4 17.73 nmol/L, T3 0.85 nmol/L, TSH 2.18 mIU/L. ② Pituitary hormones: Cortisol (08:00) 29.59 nmol/L, Cortisol (16:00) 35.66 nmol/L, Cortisol (00:00) 19.55 nmol/L, adrenocorticotropic hormone 21.65 pg/mL, prolactin 43.30 mIU/L, LH 0.88 mIU/L, and FSH 1.28 mIU/L. The results of other laboratory tests are shown in **Table 1**. In addition, pituitary enhanced MRI suggested an enlarged sella turcica, filled with cerebrospinal fluid, compressed pituitary becoming flattened, and a right deviation of the pituitary stalk (**Figure 1**). When we combined all this information, including hormone levels, severe postpartum hemorrhage, lack of lactation and amenorrhoea, and an MRI suggesting an empty sella turcica, we diagnosed the patient with Sheehan’s syndrome. Following consultation with the endocrinology department, the patient underwent a staged treatment plan. Initially, intravenous methylprednisolone was administered at a dose of 40 mg per day After three days of treatment, the patient exhibited significant improvement in symptoms of excessive crying, excessive worry, and suicidal ideation. Following this, the patient’s medication regimen was then transitioned to oral hydrocortisone (20mg in the morning and 10mg in the afternoon), in conjunction with levothyroxine sodium at 50ug per day. Following a period of seven days, the patient’s condition was stabilized, and subsequent pituitary hormone tests revealed a significant improvement in cortisol and adrenocorticotropic hormone levels. Specifically, morning cortisol levels increased to 233.44 nmol/L, while adrenocorticotropic hormone reached 4.15 pmol/L. The patient was subsequently discharged on a maintenance dose of oral hydrocortisone at 20mg per day.

**Table 1:** Laboratory results of pituitary hormone and other relevant tests.

Parameters	Value	Reference range
Hematology		
White blood cells (×10^9^/L)	5.95	3.5-9.5
Hemoglobin (g/L)	116	115-150
Platelets (×10^9^/L)	190	125-350
Serum Biochemistry		
Plasma sodium (mmol/L)	133.2	137.0-147.0
Serum calcium (mmol/L)	2.40	2.08-2.60
Serum phosphorus (mmol/L)	1.46	0.81-1.45
Serum protein (g/L)	74.2	65.0-85.0
Serum albumin (g/L)	41.0	40.0-55.0
Hormone Profile		
Total T3 (nmol/L)	0.85	1.01-2.48
Total T4 (nmol/L)	17.73	69.97-152.52
Free T3 (pmol/L)	3.83	3.09-7.42
Free T4 (pmol/L)	<3.20	7.64-16.03
TSH (mIU/L)	2.18	0.49-4.91
GH (ng/mL)	0.04	0.01-3.61
FSH: (IU/L)	1.28	16.74-113.59
LH (IU/L)	0.88	10.87-58.64
Prolactin (mIU/L)	43.30	58.09-416.37
Estradiol (pmol/L)	55.10	55.1-92.1
Cortisol (nmol/L)	29.50	185-624
ACTH (pg/ml)	21.65	7.2-63.4

TSH, Thyroid-stimulating hormone; GH, Growth hormone; FSH, Follicle-stimulating hormone; LH, Luteinizing hormone; ACTH, Adrenocorticotrophic hormone.

Following a one-month period, a telephone follow-up was conducted with the patient. It was reported a noticeable improvement in energy levels and physical strength, as well as increased mental agility. The patient also stated that her sleep requirements had decreased significantly. Furthermore, the suicidal thoughts that had previously troubled her had completely disappeared. However, she still expressed concerns about her daughter’s marital issues and ongoing anxiety regarding her family’s financial situation.

## Discussion

We hereby present a case of a 50-year-old female patient suffering from Sheehan’s syndrome who exhibited symptoms of delayed-onset anxiety and depression 23 years following childbirth. Following the administration of targeted hormone replacement therapy (glucocorticoids and thyroid hormones), a marked improvement in her symptoms was observed.

Sheehan’s syndrome is characterized by pituitary necrosis resulting from ischemic injury triggered by postpartum hemorrhage. Its pathogenesis involves the unique physiological vulnerability of the pituitary gland during pregnancy and multiple pathological factors (9, 10). Under estrogen stimulation, the hyperplasia of pituitary lactotrophs leads to glandular hypertrophy, increasing metabolic demand and making the gland more susceptible to hypoperfusion injury during postpartum hemorrhage. Ischemia not only directly induces necrosis but is also exacerbated by vasospasm, coagulation abnormalities, and potential autoimmune responses targeting pituitary antigens. These mechanisms collectively contribute to the progressive development of hypopituitarism. Sheehan’s syndrome presents with a variety of symptoms. Amenorrhea, agalactorrhea, cold intolerance, constipation, and dry skin are among the most common manifestations. Furthermore, this syndrome can also be associated with anxiety and depression (6–8). However, because of the rarity of anxiety and depression symptoms, they are often overlooked. Qadri et al. reported a case of a 45-year-old female patient with Sheehan’s syndrome presenting with major depressive disorder (6). Reddy et al. described a case of Sheehan’s syndrome manifesting as an extremely rare psychiatric condition (7). Similarly, Tıkır et al. documented a married patient with Sheehan’s syndrome who exhibited symptoms of mental disorder (8). Furthermore, there are few case reports suggesting an association between psychiatric symptoms and Sheehan’s syndrome.

The etiology of anxiety and depression in Sheehan’s syndrome remains unclear. Hormonal deficiencies disrupt the normal regulatory function of the hypothalamic-pituitary-adrenal (HPA) axis. Low cortisol levels have been demonstrated to impair the body’s stress response and to directly affect the function of emotional regulation centers, such as the hippocampus and prefrontal cortex, by interfering with the synthesis and release of neurotransmitters like serotonin and norepinephrine. This, in turn, has been shown to contribute to symptoms of low mood, anxiety, and depression (11–14). Concurrently, a low thyroid hormone state has been shown to exacerbate central nervous system dysfunction by affecting brain cell energy metabolism and neuronal excitability (13, 15). In our patient, significant declines in the secretion of multiple hormones, including thyroid hormone and cortisol, may provide a biological basis for the presence of anxiety and depression. Furthermore, research has indicated that growth hormone (GH) and its dependent insulin-like growth factor 1 (IGF-1) play pivotal roles in preserving neuronal survival, promoting neural repair, and sustaining cognitive function. Deficiencies in these factors have been shown to impede the nervous system’s capacity to repair and adapt to local injuries and chronic stress, consequently exacerbating cognitive and emotional dysfunction (16, 17). Although the patient’s serum GH level was within the normal range (0.04 ng/mL; reference value: 0.01-3.61 ng/mL), it is worth noting that GH secretion is pulsatile and influenced by a variety of factors, and a basal GH measurement cannot be used for the evaluation of GH sufficiency per se (18, 19). Dynamic tests (e.g., insulin tolerance tests) would provide a more definitive assessment of GH deficiency. However, such tests are not available in our clinical setting. In addition, although IGF-1 is a valuable marker of GH deficiency, serum IGF-1 levels were not measured because the patients’ GH levels and clinical presentation did not strongly suggest GH deficiency. Meanwhile, our patient also faced psychosocial stressors such as financial pressure and concerns about her child’s marriage. In addition to hormonal deficiencies, psychosocial stressors may have exacerbated her psychiatric symptoms. Research indicates that patients with chronic illnesses are particularly vulnerable to psychosocial stress, which can worsen anxiety and depression (20). These external stressors persistently activate the HPA axis, leading to abnormal cortisol regulation and further disrupting neurotransmitter balance, thereby synergistically interacting with endocrine dysfunction to promote the onset and exacerbation of emotional disorders such as anxiety and depression (21). Moreover, it has been documented that pituitary ischemic necrosis may instigate an autoimmune process (22, 23). The release of sequestered antigens due to tissue necrosis may precipitate pituitary autoimmunity and result in delayed hypopituitarism in Sheehan’s syndrome.

Although there is a high degree of similarity in the psychiatric manifestations of dysthymia co-occurring with Sheehan’s syndrome and generalized anxiety disorder with depressive symptoms (24, 25), our findings provide strong support for the diagnosis of Sheehan’s syndrome. The patient exhibited a medical history marked by severe postpartum hemorrhage, subsequently followed by agalactorrhea and amenorrhea, Concurrently, the patient’s laboratory results indicated multiple pituitary hormone deficiencies, and magnetic resonance imaging (MRI) revealed an empty sella. These findings provide substantial corroboration for the diagnosis of Sheehan’s syndrome. Studies have shown that patients with hypopituitarism, including those with Sheehan’s syndrome, frequently manifest psychiatric symptoms, and hormone replacement therapy can alleviate these symptoms (7, 26, 27). Replacement therapy with thyroid hormones and glucocorticoids has been demonstrated to enhance energy levels and mood in patients; however, achieving complete symptom resolution may necessitate an extended duration of therapy or the incorporation of supplementary psychological interventions (28).

The diagnosis of Sheehan’s syndrome is fraught with numerous challenges, chief among them being the limited awareness of the condition among clinicians. Sheehan’s syndrome can manifest with prominent psychiatric symptoms, yet the underlying endocrine etiology is frequently overlooked. The absence of standardized screening protocols and guidelines further complicates diagnosis, underscoring the importance of targeted screening in patients with a history of postpartum hemorrhage. In resource-limited settings, the unavailability of advanced imaging techniques and dynamic hormone testing exacerbates the risk of missed diagnoses. Consequently, integrating endocrine assessments into the diagnostic workflow for at-risk populations is essential to enhance early detection of Sheehan’s syndrome. To address these challenges, educational initiatives targeting endocrinologists, psychiatrists, and general practitioners should emphasize the clinical association between postpartum hemorrhage, amenorrhea, and psychiatric symptoms. Multidisciplinary collaboration holds significant potential to improve early recognition rates. Furthermore, patients presenting with unexplained chronic mood disorders should undergo routine endocrine evaluations, including assessments of cortisol, thyroid hormone, and growth hormone levels. While basal hormone testing and magnetic resonance imaging (MRI) remain fundamental diagnostic tools, dynamic testing (e.g., insulin tolerance tests) and insulin-like growth factor 1 (IGF-1) assessment should be prioritized over isolated basal hormone measurements. Dynamic testing offers a more accurate evaluation of pituitary reserve function, thereby providing a more reliable basis for diagnosing Sheehan’s syndrome.

In our case, although the diagnosis of anxiety and depression was made by a psychiatrist in the outpatient setting using DSM-5 criteria, it is recognized that the lack of standardized scale-based assessments during hospitalization limited the quantitative evaluation of mood changes. It is therefore recommended that future studies integrate clinical judgment with objective tools to enhance the validity of such diagnoses in patients with Sheehan’s syndrome.

A further limitation of the present study is that the function of the GH-IGF-1 axis was not systematically evaluated. Despite the presence of normal basal GH levels, the absence of dynamic testing and IGF-1 detection may underestimate the possibility of GH deficiency. Future studies should include IGF-1 measurements and dynamic testing to further explore the role of GH in the neuropsychiatric manifestations of Sheehan’s syndrome.

## Conclusion

The rarity of cases in which patients diagnosed with Sheehan’s syndrome present with anxiety and depression poses a significant clinical diagnostic challenge, necessitating meticulous clinical assessment and swift therapeutic intervention. The atypical presentation of patients necessitates heightened vigilance on the part of clinicians for the presence of endocrine disorders, particularly in patients with a protracted course of the disease. Future research endeavors should concentrate on the formulation of precise diagnostic and therapeutic guidelines for patients with Sheehan syndrome, with a view to improve enhancing the prognosis of this demographic.

## Data Availability

The raw data supporting the conclusions of this article will be made available by the authors, without undue reservation.

## References

[1] Kristjansdottir HL Bodvarsdottir SP Sigurjonsdottir HA . Sheehan’s syndrome in modern times: a nationwide retrospective study in Iceland. Eur J Endocrinol. (2011) 164:349–54. doi: 10.1530/EJE-10-1004 21183555

[2] Gei-Guardia O Soto-Herrera E Gei-Brealey A Chen-Ku CH . Sheehan syndrome in Costa Rica: clinical experience with 60 cases. Endocr Pract. (2011) 17:337–44. doi: 10.4158/EP10145.OR 21041170

[3] Saxena S Verma V Samadarshi S Dorji T Muthukrishnan J . Delayed Sheehan’s syndrome diagnosed during the evaluation of secondary infertility: A case report. Clin Case Rep. (2024) 12:e8521. doi: 10.1002/ccr3.8521 38344342 PMC10853049

[4] Soresi M Brunori G Citarrella R Banco A Zasa A Di Bella G . Late-onset Sheehan’s syndrome presenting with rhabdomyolysis and hyponatremia: a case report. J Med Case Rep. (2013) 7:227. doi: 10.1186/1752-1947-7-227 24083446 PMC3843569

[5] Rabee’ H Braik T Alnatour R Shamlawi A Rashed A . Sheehan’s syndrome unveiled after decades without a diagnosis: A case report. SAGE Open Med Case Rep. (2023) 11:2050313X231209685. doi: 10.1177/2050313X231209685 PMC10624101 37927363

[6] Qadri MI Mushtaq MB Qazi I Yousuf S Rashid A . Sheehan’s syndrome presenting as major depressive disorder. Iran J Med Sci. (2015) 40:73–6. PMC4300485 25648343

[7] Reddy MS Nahar A Thippeswamy H Kumar CS . Psychosis as a late manifestation of Sheehan’s syndrome. Asian J Psychiatr. (2017) 25:228–30. doi: 10.1016/j.ajp.2016.12.003 28262158

[8] Tıkır B Göka E Aydemir MÇ Gürkan Ş . Psychotic disorder and sheehan’s syndrome: etiology or comorbidity?: A case report. Turk Psikiyatri Derg. (2015) 26:142–45. 26111291

[9] Karaca Z Kelestimur F . Sheehan syndrome: a current approach to a dormant disease. Pituitary. (2025) 28:20. doi: 10.1007/s11102-024-01481-1 39863703 PMC11762620

[10] Diri H Karaca Z Tanriverdi F Unluhizarci K Kelestimur F . Sheehan’s syndrome: new insights into an old disease. Endocrine. (2016) 51:22–31. doi: 10.1007/s12020-015-0726-3 26323346

[11] Peters AT Van Meter A Pruitt PJ Briceño EM Ryan KA Hagan M . Acute cortisol reactivity attenuates engagement of fronto-parietal and striatal regions during emotion processing in negative mood disorders. Psychoneuroendocrinology. (2016) 73:67–78. doi: 10.1016/j.psyneuen.2016.07.215 27474908 PMC5048542

[12] Chatterjee SS Mitra S Mallik N Ghosal M . Even very low dose hydrocortisone might precipitate psychosis in Sheehan’s syndrome: A need for caution. J Neurosci Rural Pract. (2016) 7:333–34. doi: 10.4103/0976-3147.176189 PMC4821961 27114684

[13] Kate S Dhanwal DK Kumar S Bharti P . Acute psychosis as a presentation of hypopituitarism. BMJ Case Rep. (2013) 2013:1–3. doi: 10.1136/bcr-2012-008516 PMC3736158 23853186

[14] Nath S Ranjan R Mohapatra D Mishra BR . Successful management of patient with Sheehan’s syndrome presenting with psychosis and catatonia. Indian J Psychol Med. (2018) 40:276–79. doi: 10.4103/IJPSYM.IJPSYM_280_17 PMC5968652 29875538

[15] Davis JD Tremont G . Neuropsychiatric aspects of hypothyroidism and treatment reversibility. Minerva Endocrinol (Torino). (2007) 32:49–65. 17353866

[16] Lynch S Merson S Beshyah SA Skinner E Sharp P Priest RG . Psychiatric morbidity in adults with hypopituitarism. J R Soc Med. (1994) 87:445–47. doi: 10.1177/014107689408700805 PMC1294682 8071912

[17] Imran SA Wilkinson M . Cognition and psychological wellbeing in hypopituitary patients. Rev Endocr Metab Disord. (2024) 25:505–12. doi: 10.1007/s11154-023-09869-3 38146042

[18] Nass R Gaylinn BD Thorner MO . The role of ghrelin in GH secretion and GH disorders. Mol Cell Endocrinol. (2011) 340:10–4. doi: 10.1016/j.mce.2011.03.021 PMC4205082 21459126

[19] Steyn FJ Huang L Ngo ST Leong JW Tan HY Xie TY . Development of a method for the determination of pulsatile growth hormone secretion in mice. Endocrinology. (2011) 152:3165–71. doi: 10.1210/en.2011-0253 21586549

[20] Scott AJ Correa AB Bisby MA Dear BF . Depression and anxiety trajectories in chronic disease: A systematic review and meta-analysis. Psychother Psychosom. (2023) 92:227–42. doi: 10.1159/000533263 37607505

[21] Aas M Vecchio C Pauls A Mehta M Williams S Hazelgrove K . Biological stress response in women at risk of postpartum psychosis: The role of life events and inflammation. Psychoneuroendocrinology. (2020) 113:104558. doi: 10.1016/j.psyneuen.2019.104558 31923613

[22] Goswami R Kochupillai N Crock PA Jaleel A Gupta N . Pituitary autoimmunity in patients with Sheehan’s syndrome. J Clin Endocrinol Metab. (2002) 87:4137–41. doi: 10.1210/jc.2001-020242 12213861

[23] Keleştimur F . Sheehan’s syndrome. Pituitary. (2003) 6:181–88. doi: 10.1023/b:pitu.0000023425.20854.8e 15237929

[24] de Silva NL Galhenage J Dayabandara M Somasundaram N . Sheehan syndrome presenting with psychotic manifestations mimicking schizophrenia in a young female: A case report and review of the literature. Case Rep Endocrinol. (2020) 2020:8840938. doi: 10.1155/2020/8840938 33343948 PMC7732407

[25] Hek K Tiemeier H Newson RS Luijendijk HJ Hofman A Mulder CL . Anxiety disorders and comorbid depression in community dwelling older adults. Int J Methods Psychiatr Res. (2011) 20:157–68. doi: 10.1002/mpr.344 PMC6878519 22547298

[26] Shoib S Dar MM Arif T Bashir H Bhat MH Ahmed J . Sheehan’s syndrome presenting as psychosis: a rare clinical presentation. Med J Islam Repub Iran. (2013) 27:35–7. PMC3592941 23483784

[27] Michalczyk J Miłosz A Soroka E . Postpartum psychosis: A review of risk factors, clinical picture, management, prevention, and psychosocial determinants. Med Sci Monit. (2023) 29:e942520. doi: 10.12659/MSM.942520 38155489 PMC10759251

[28] Slagboom T Deijen JB Van Bunderen CC Knoop HA Drent ML . Psychological well-being and illness perceptions in patients with hypopituitarism. Pituitary. (2021) 24:542–54. doi: 10.1007/s11102-021-01131-w PMC8270855 33606176

